# ABO Blood Type and Personality Traits in Healthy Japanese Subjects

**DOI:** 10.1371/journal.pone.0126983

**Published:** 2015-05-15

**Authors:** Shoko Tsuchimine, Junji Saruwatari, Ayako Kaneda, Norio Yasui-Furukori

**Affiliations:** 1 Department of Neuropsychiatry, Graduate School of Medicine, Hirosaki University, Hirosaki, Japan; 2 Division of Pharmacology and Therapeutics, Graduate School of Pharmaceutical Sciences, Kumamoto University, Kumamoto, Japan; National Center of Neurology and Psychiatry, JAPAN

## Abstract

There is no scientific consensus that a relationship exists between the ABO blood group and personality traits. However, a recent study hypothesized that the dopamine beta-hydroxylase (*DBH*) gene is in linkage with the *ABO* gene. The sample population consisted of 1,427 healthy Japanese subjects who completed the Temperament and Character Inventory (TCI). Each subject’s ABO blood type was determined by genotyping the rs8176719 and rs8176746 *ABO* gene single-nucleotide polymorphisms (SNPs) using a TaqMan genotyping assay. The relationships between the six *ABO* genotypes or four ABO phenotypes and personality traits were examined using a multivariate analysis of covariance (MANCOVA), controlling for age and sex. The MANCOVA data showed a significant difference in TCI scores among the *ABO* genotype groups (F [7, 1393] = 3.354, *p* = 0.001). A subsequent univariate analysis showed a significant difference in the mean scores for Persistence among the genotype groups (*F* = 2.680, *partial η^2^* = 0.010, *p* = 0.020). Similarly, dividing the ABO blood type into four phenotypes revealed a significant difference among the phenotype groups (F [7, 1397] = 2.529, *p* = 0.014). A subsequent univariate analysis showed a significant difference among the phenotype groups in the mean scores for Persistence (*F* = 2.952, *partial η^2^*= 0.006, *p* = 0.032). We observed a significant association between ABO blood group genotypes and personality traits in a large number of healthy Japanese subjects. However, these results should be regarded as preliminary and should be interpreted with caution because it is possible that the association between ABO blood group genotype and the Persistence trait is relatively weak.

## Introduction

Personality traits influence many aspects of normal and pathological behaviors [1–5]. Temperament traits, the most basic part of personality, have been correlated with neurotransmitter systems and are genetically controlled [2]. In the last decade, a large number of studies have focused on the detection of genetic variants associated with specific temperament traits, and numerous findings have been reported [6][7]. Although a consensus has not been reached, many genes show reproducible associations with personality traits, including the current consensus that personality is approximately 50% heritable [8][9].

ABO blood group type is genetically predetermined and easily identified, which has led to its use as a biological marker to assess the influence of genetic factors on personality in various ethnic groups [10–19]. Although researchers sought to uncover blood type-related personality factors prior to 2000, the results were inconsistent [12–18]. Since 2000, a few published studies have examined possible associations between blood group and personality traits using the NEO Personality Inventory to assess the Big Five personality traits, which represent five broad dimensions of personality; however, they failed to find any associations [10][11][19][20]. In addition, a more recent study found no significant association between ABO blood groups and personality traits [21].

However, a scientific hypothesis concerning ABO blood groups and personality traits was recently proposed by Hobgood [22][23]. Dopamine beta-hydroxylase (DBH) is known to catalyze the conversion of dopamine to norepinephrine, and the *DBH* gene is in tight linkage with the *ABO* gene on chromosome 9q34 [24][25]. In addition, the *DBH* gene contains a functional single-nucleotide polymorphism (SNP; rs1611115). The most common allele, rs1611115C, is the predominant determinant of high DBH activity, whereas rs1611115T is the low-activity allele [26]. Personality trait research has found that low-activity DBH is associated with the impulsiveness trait, whereas high-activity DBH is associated with sensation seeking [27][28]. The ABO group B marker rs8176746 and the low-activity *DBH* allele rs1611115T exhibit similar frequency distributions among HapMap populations. For these reasons, and considering the known effects of catecholamine on behavior, Hobgood [22][23] suggested that linkage between *DBH* and *ABO* may explain the associations between personality traits and ABO blood type.

There are several lines of evidence that ABO blood type is associated with various diseases, including cardiovascular disease, cancer, and stress response-related immune disease [29]. There are also reports that specific personality traits, such as depression and anxiety, may be associated with these diseases [30–32]. Therefore, it is possible that ABO blood type is also associated with personality traits.

We evaluated two SNPs, rs8176719 and rs8176746, as markers of the *ABO* genotype in this study. The rs8176719 SNP indicates *O*-allele-specific 261delG, and the rs8176746 SNP determines the galactose specificity of the encoded A/B transferases and, thus, the expression of A and B antigens on erythrocytes [33]. Many previous studies examining these two SNPs have reported associations between ABO blood type and various diseases, including the ‘blood type diet’, cardiometabolic risk, susceptibility to severe *Plasmodium falciparum* malaria, and pancreatic cancer [34–36].

Based on the evidence mentioned above, it is assumed that to understand the effect of the ABO blood types on personality traits, it is necessary to investigate not only ABO phenotypes but also *ABO* genotypes. The aim of this study was to evaluate possible associations between ABO phenotypes and genotypes and personality traits in a large sample of healthy Japanese subjects.

## Subjects and Methods

### Subjects

In total, 1,572 healthy Japanese medical school students and medical staff were recruited to participate in this study. Two well-trained psychiatrists used the non-patient version of the Structured Clinical Interview of the Diagnostic and Statistical Manual of Mental Disorders (DSM-IV) to assess all of the subjects. Subjects with neurological axis DSM-IV psychiatric disorders were excluded. This study was approved by the Ethics Committee of the Hirosaki University School of Medicine. Written informed consent, using an Ethics Committee-approved consent document, was obtained from all the subjects; a verbal explanation was also provided. The original signed documents were filed as research records.

A subset of subjects were excluded from the analysis because they declined to complete the TCI or because complete genotype results could not be obtained. Ultimately, 1,427 subjects (849 males and 578 females) with complete data sets were included in the analysis. The age range of these subjects was 18–69 years, with a mean (± standard deviation [SD]) of 28.2 (±9.0) years. The Japanese version of the TCI was used to assess personality traits [37].

### DNA analysis

Genomic DNA was extracted from 5 ml of peripheral blood using a DNA purification kit (QIAGEN, Germany). Genotypes for the two SNPs of the *ABO* gene (SNP ID: rs8176719 and rs8176746) were assessed using the TaqMan allele-specific assay method (Applied Biosystems, Inc. [ABI], Foster City, CA, USA) according to the manufacturer’s instructions. We also examined the *DBH* gene to investigate whether the *ABO* gene is in linkage with the *DBH* gene. ABI’s two dual-labeled probes center on SNPs that differ in sequence only at the 1-bp polymorphism. The probes were labeled with 5’ reporter fluorophores (VIC or 6-FAM) and a 3’ quencher. The rs8176719 variant encodes the exon 6 261G deletion that results in the common *O* allele, whereas rs8176746 encodes exon 7 C796A, which is 1 of the 7 standard *ABO* variants that distinguish *A* alleles from *B* alleles. The SNP rs1611115 is located in the promoter region of the human *DBH* gene (C-T change at nucleotide position −1021) and functionally controls the DBH activity. Commercial probes and primers were used for rs8176746 and rs1611115 (assay IDs: C__25610772_20 for rs8176746 and C__2535786_10 for rs1611115, Applied Biosystems), whereas custom probes and primers were specifically designed for rs8176719. Genotyping for rs8176719 was performed using PCR with 5’-CCATGTGCAGTAGGAAGGATGT-3’ and 5’-TGCCCTCCCAGACAATGG-3’ primers and 6FAM-TCGTGGTGACCCCTT/VIC-CCTCGTGGTACCCCTT probes. All of the assays were conducted in 96-well PCR plates using the 7500 Fast Real-Time PCR System with the corresponding 7500 Fast System SDS software (Applied Biosystems). The amplification reactions were performed in duplicate with 30 ng (1 μl) of template DNA, 2× TaqMan Universal Master Mix buffer (Applied Biosystems), and 40× primer and probe mix (Applied Biosystems). Thermal cycling was performed under the following conditions: denaturation at 95°C for 20 sec, followed by 40 cycles of 95°C for 3 sec and 62°C for 30 sec. To validate the assay, the quality of the genotyping data was routinely assessed statistically by testing for Hardy-Weinberg equilibrium. In addition, a random sampling of 5% of subjects was retyped, and the data were compared to the hemagglutination phenotype results (A, B, AB, and O), which showed 100% concordance.

### Temperament and Character Inventory (TCI)

Personality was assessed using the Temperament and Character Inventory (TCI) [2], a widely used measure of personality traits. The TCI, which consists of 240 items, is used to assess individual differences in the basic dimensions of the biosocial model of personality. This model is based on the assumption that part of an individual's personality is heritable. Cloninger hypothesized that personality is composed of traits that are heritable and stable throughout life, as well as traits that are influenced by socio-cultural learning and that mature throughout life. The TCI consists of seven dimensions, including three temperament dimensions and four character dimensions. Of the temperament dimensions, which include Novelty Seeking, Harm Avoidance, Reward Dependence, and Persistence, three have been hypothesized to be related to monoamine neurotransmitters: Novelty Seeking is hypothesized to be related to dopaminergic activity, Harm Avoidance to serotonergic activity and Reward Dependence to noradrenergic activity. Novelty Seeking relates to exploratory behaviors and activation in response to novel stimuli; Harm Avoidance refers to an individual's inclination for behavioral inhibition when facing potentially dangerous stimuli and the tendency to anticipate negative effects; Reward Dependence concerns relational and affective skills and dependent attitudes; and Persistence characterizes the traits of industriousness, diligence and stability despite frustration and fatigue. Character consists of three dimensions: Self-directedness, Cooperativeness and Self-transcendence. Self-directedness reflects the individual's levels of autonomy, reliability and maturity; Cooperativeness is related to social skills, including support, collaboration, and partnership; and Self-transcendence denotes an aptitude for mysticism, religion and idealism.

### Statistical analysis

A multivariate analysis of covariance (MANCOVA) was performed with TCI scores as the dependent variables and *ABO* genotypes (*AA*, *AO*, *BB*, *BO*, *OO*, and *AB*), phenotypes (A, B, O, and AB), and the covariates of age and sex. Dummy variables were used for sex (male = 0, female = 1). Before the MANCOVA analyses, the normality of distributions was checked for each TCI score, genotype, and phenotype subgroup; these showed normal distributions. In all the MANCOVAs, we used Roy’s largest root statistic with an alpha level of 0.05. The data were analyzed using PASW Statistics for Windows software, version 18.0.0 (SPSS, Inc., Chicago, IL, USA). Pairwise linkage disequilibrium was evaluated using the SNPAlyze V6.0 Standard software program (Dynacom Co. Ltd, Chiba, Japan).

## Results

Tables 1 and 2 show the genotype and phenotype distributions of the ABO group, as well as the mean scores and standard deviations for the seven TCI factors for each genotype and phenotype. The allele groups showed the following frequencies: *A* alleles (26.7%), *B* alleles (17.7%), and *O* alleles (55.6%). These *ABO* gene allele and phenotype frequencies were similar to those observed in other samples from Japanese populations [38]. The genotype distribution was consistent with Hardy-Weinberg equilibrium (*p* = 0.46). A significant but small to moderate degree of linkage disequilibrium was detected between ABO rs81746746 and DBH rs1611115 (|D'| = 0.4009, *p* = 0.005819) and between ABO rs8176719 and DBH rs1611115 (|D'| = 0.2131, *p* = 0.006529), and a large linkage disequilibrium was observed between ABO rs8176749 and 8176719 (|D'| = 1, *p* = 7.71×10^-66^).

**Table 1:** MANCOVA for TCI scores and *ABO* genotype groups (mean ± SD).

*ABO* genotype	rs8176746 × rs8176719 Genotype	N (%)	Novelty Seeking	Harm Avoidance	Reward Dependence	Persistence	Self-directedness	Cooperativeness	Self-transcendence
*AA*	*CC × GG*	99 (6.9)	21.8±5.2	18.3±6.4	15.4±3.1	4.9±1.8	28.0±6.0	28.7±6.2	9.9±5.2
*AO*	*CC × GD*	438 (30.7)	21.9±5.2	18.6±6.4	15.1±3.2	4.5±1.9	28.0±6.6	28.1±5.3	9.2±4.8
*BB*	*AA × GG*	47 (3.3)	22.3±5.4	17.1±6.0	14.9±3.4	4.6±2.0	27.6±6.9	27.6±5.9	10.3±5.6
*BO*	*AC × GD*	284 (19.9)	22.0±5.3	19.0±6.3	14.7±3.3	4.2±1.8	28.2±6.8	27.8±5.5	9.4±4.7
*OO*	*CC × DD*	433 (30.3)	22.1±5.1	18.9±6.1	15.0±3.1	4.3±1.8	28.2±6.4	28.4±5.0	9.4±4.4
*AB*	*AC × GG*	126 (8.8)	21.4±4.4	19.8±5.9	14.9±3.0	4.4±1.9	28.1±6.9	27.5±5.3	9.5±5.2
*F*			0.491	1.303	0.875	2.683	0.060	0.992	0.70
*Partial η* ^*2*^			0.002	0.005	0.003	0.010	0.000	0.004	0.003
*P-value*			0.784	0.260	0.497	**0.020** ^**¶**^	0.998	0.421	0.622

Comparison of the TCI scores among the six genotype groups including age and sex as covariates.

The rs 8176719 alleles are exon 6 261G ("G") and 261delG ("D"). The rare genotypes *AA* × *DD*, *AC* × *DD*, and *AA* × *GD* did not occur in our samples.

^¶^There was a significant difference between the ABO blood types and Persistence scores. Post hoc analysis showed that *AA* genotype group had higher Persistence scores than *BO* and *OO* genotype group (*p* = 0.017 and *p* = 0.045, respectively; Bonferroni correction).

**Table 2:** MANCOVA for TCI scores and the ABO phenotype groups (mean ± SD)

ABO phenotype	N (%)	Novelty Seeking	Harm Avoidance	Reward Dependence	Persistence	Self-directedness	Cooperativeness	Self-transcendence
A (allele *AA*, *AO*)	537 (37.6)	21.9±5.2	18.6±6.4	15.1±3.2	4.6±1.9	28.0±6.5	28.2±5.5	9.3±4.9
B (allele *BB*, *BO*)	331 (23.2)	22.1±5.3	18.7±6.3	14.7±3.3	4.3±1.8	28.1±6.8	27.8±5.6	9.5±4.8
O (allele *OO*)	433 (30.3)	22.1±5.1	18.9±6.1	15.0±3.1	4.3±1.8	28.2±6.4	28.4±5.0	9.4±4.4
AB (allele *AB*)	126 (8.8)	21.4±4.4	19.8±5.9	14.9±3.0	4.4±1.9	28.1±6.9	27.5±5.3	9.5±5.2
*F*		0.795	1.293	0.800	2.952	0.093	1.202	0.064
*Partial η* ^*2*^		0.002	0.003	0.002	0.006	0.000	0.003	0.000
*P-value*		0.496	0.275	0.494	**0.032** ^**¶**^	0.964	0.308	0.979

Comparison of the TCI scores among the four phenotype groups including age and sex as covariates.

^¶^There was a significant difference between the ABO phenotypes and Persistence scores. Post hoc analysis showed that blood type A group had higher Persistence scores than B and O groups (*p* = 0.009 and *p* = 0.018, respectively; Bonferroni correction).

The MANCOVA analysis of the TCI scores revealed a significant difference between the genotype groups (F [7, 1393] = 3.354, *p* = 0.001). A subsequent univariate ANCOVA showed a significant difference in the mean scores for Persistence among the *ABO* genotype groups (*F* = 2.680, *partial η* ^*2*^ = 0.010, *p* = 0.020; Table 1), with the post hoc analysis showing that subjects carrying the *AA* genotype had significantly higher Persistence scores than did those carrying the *BO* and *OO* genotypes (*p* = 0.017 and *p* = 0.045, respectively; Bonferroni correction). No significant relationship was observed between *ABO* genotype and age or sex.

Similarly, a significant difference was observed between phenotype groups (F [7, 1397] = 2.529, *p* = 0.014) when the ABO blood type was divided into four phenotypes. A subsequent univariate ANCOVA showed a significant difference among the ABO phenotype groups in the mean scores for Persistence (*F* = 2.952, *partial η* ^*2*^ = 0.006, *p* = 0.032; Table 2). A post hoc analysis showed that the blood type A group had higher Persistence scores than the B and O groups (*p* = 0.009 and *p* = 0.018, respectively; Bonferroni correction). There were no significant interactions between ABO phenotype group and age or sex.

No significant association was observed between *DBH* rs1611115 and any of the TCI scores (data not shown).

## Discussion

In this study, we investigated possible associations between ABO phenotype and genotype and personality traits based on Hobgood’s hypothesis [22][23]. The results of this study showed significant differences in Persistence scores according to both genotype and phenotype. Subjects with the *A* allele had higher Persistence scores than subjects with *B* or *O* alleles in both case genotype and case phenotype. However, these results are inconsistent with those of previous studies [10–12][15][16][19][20][21].

Previous studies have demonstrated only associations between ABO phenotype (blood type) and personality traits. However, according to Hobgood’s hypothesis [22][23], it is essential to analyze the relationship between personality traits and both the *ABO* genotype and the ABO phenotype. The strength of our research was the evaluation of both the *ABO* genotype and the ABO phenotype and the identification of linkage disequilibrium between the *ABO* and *DBH* genes. Our results revealed a significant difference in Persistence scores, although the effect size was small (partial *η* ^2^ = 0.010, MANCOVA). In addition, we also found a significant, small to moderate linkage disequilibrium between the *ABO* and *DHB* genes. These findings appear to support the previous hypothesis that the *ABO* and *DBH* genes are in linkage, and ABO group B subjects with low DBH activity show low Persistence, whereas ABO group A and O subjects with high DBH activity show high Persistence [22][23]. However, our findings indicated the same effect of *B*- and *O*-alelles opposite to the *A*-allele effect on Persistence. Hence, it follows that these results could not explain only a fraction of the previous hypothesis. It is still unclear why the *A*-allele has an effect opposite to the *B*- and *O*-alleles on Persistence because there is no evidence of the influence of the *ABO* gene on personality traits so far. There is a report that the *ABO* gene is possibly linked to other catecholamine genes such as catechol-O-methyltransferase (*COMT*) and monoamine oxidase A (*MAOA*) through certain research findings and HapMap population frequency distributions [22]. Therefore, it is possible that the difference between the influence of the *A* allele and of the *B*- and *O*-alleles on Persistence might be indirect through a complex interaction between the *ABO* gene and the catecholamine genes.

Persistence was originally proposed as a subscale of reward dependence, although the specific brain systems involved in this process have yet to be identified. Some association studies have revealed links between the Persistence trait and dopamine transporters and receptors [39–42]. In addition, we previously reported that a homozygous *MAOA* gene polymorphism (*MAOA-VNTR*) for a high-activity allele was associated with significantly higher Persistence scores compared with the low-activity allele [43]. Moreover, Rinieris et al. [44] reported that obsessive-compulsive disorder displayed genetic linkage with the ABO blood type A, although this result was not in agreement with other studies [45]. Based on these results, the ABO blood group may have an influence on personality traits, particularly the association between ABO group A and Persistence. This association may be related to neurotransmitter activity, such as dopaminergic activity, although the molecular biological mechanisms underlying the role of blood type in personality differences remain unknown.

There is a long tradition of popular interest in and the use of ABO blood group typing to understand individual personalities in Japan and other East Asian countries, where this theory is commonly accepted. In particular, the Japanese ABO blood group personality theory proposes that blood types A, B, O, and AB are associated with personality traits related to seriousness and enthusiasm, being easily bored, mildness, and individualism, respectively. These conclusions regarding personality and ABO type appear partly congruent with our results: subjects carrying the *AA* genotype showed high Persistence scores, whereas those carrying the *BO* or *OO* genotype showed low Persistence scores.

Additionally, we investigated the relationship between ABO phenotype and personality traits using methods similar to those of previous studies, and we identified a significant difference between the mean scores for Persistence according to both ABO phenotype and genotype groups (*Partial η* ^*2*^ = 0.006, MANCOVA). These data are inconsistent with previous research showing no associations between ABO blood type and personality traits [10][11][20]. To detect small effects of genotypes or alleles on phenotypes, such as personality traits, it is necessary to use a large sample size. Previous studies have employed small sample sizes and hence may have lacked sufficient power to detect an association between ABO blood type and personality traits. However, most recent studies have also concluded that there is no relationship between blood type and personality; these studies included a large sample size in Taiwan [19] and large-scale survey data in Japan and the US [21]. This discrepancy might be due to studies using different personality assessment scales (TCI vs NEO PI-R) or not using a personality questionnaire typically used in psychology. The TCI is appropriate for studying associations between genetic polymorphisms and personality traits because genetic factors influence the TCI personality dimensions. In the present study, we found a small but significant difference between ABO blood type and personality traits, as measured by TCI, in a relatively large number of subjects.

ABO blood types and personality traits have been studied by both mainstream scientists and pseudoscientific groups. For example, in the Nazi era, type B was considered an indicator of lower instincts, which the Nazis thought to be frequently present in Asian and Jewish people. This belief was part of the propaganda of racism and pseudoscience that marked that regime. However, larger amounts of data may reveal that the ratios of various personality traits linked to the ABO blood types, which may reflect themselves in the structure of a society, are possible future candidates for analyses of social structure and relationships among societies. This future, truly scientific study of ABO blood group antigens will undoubtedly reveal the complexity of these patterns. In addition to population stratification based on founder effects and migration patterns, the intrinsic effects of the presence or absence of the A and B antigens’ effects on the cell membrane, and linkage with the catecholamine gene *DBH*, other genes near the *ABO* locus could play a role in the *ABO* linkages with personality and illnesses. The early-onset torsion dystonia gene (*DYT1*), which encodes torsinA, an ATPase, has been found to be associated with dystonia and attention deficit disorder [46], as have neighboring *DBH* alleles. Other candidate genes near the *ABO* locus include genes for the pregnancy-associated plasma protein (PAPPA), microcephaly, thrombotic thrombocytopenic purpura, acute hepatic porphyria and susceptibility to lead poisoning, amyloidosis, juvenile amyotrophic lateral sclerosis, hypophosphatemic rickets, COX deficiency, tuberous sclerosis, lymphangioleiomyomatosis, muscular dystrophy, Ehlers-Danlos connective tissue disease, aortic valve disease, susceptibility to colorectal cancer, and longevity [47].

This study had several limitations. First, we did not analyze potentially influential gene SNPs, such as *MAOA*, or *COMT*; therefore, we could not conclude whether ABO blood type affected personality traits through linkage with neurotransmitter-related genes. Second, the subjects were recruited from medical students and hospital staff, who may not sufficiently represent the general population. Uncontrolled socio-demographic factors may have affected our results, although the TCI scores in the present study were comparable to scores in previous studies conducted among young adult populations in Japan [48]. Third, this study did not consider the roles of some environmental factors, such as stressful life conditions, parenting style, socio-economic status, and season of birth, although personality traits represent complex phenotypes caused by gene × environment interactions. Recent twin and adoption studies have suggested that individual differences in personality traits result from a combination of genetic and environmental factors [49]. Forth, the Japanese races are characterized by a very low type B frequency and a relatively high type A frequency. To examine the effect of the *BB* genotype on personality traits, we would need to increase the sample size or conduct a replication study using a high-frequency type B population, such as an Indian or Chinese population. Forth, because the personality assessment was based on self-reports, it was not an objective measurement. Additional investigations are thus warranted to overcome the limitations of these existing combinatorial approaches and to examine ABO-neurotransmitter interactions related to personality traits. Finally, although we conducted this study based on the hypothesized linkage between the *ABO* and *DBH* genes, there were no significant differences in personality traits among *DBH* rs1611115 alleles. That is, the results indicated that an association between the *ABO* gene and personality traits could not be explained only by the hypothesis. The mechanisms underlying the relationship between the *ABO* gene and personality traits remain unclear due to the limited number of relevant studies. In view of these preliminary findings, the validation of Hobgood's hypothesis requires further study.

## Conclusion

Although associations between ABO blood type groups and personality traits have been treated as pseudoscience, we observed a significant association between ABO blood group genotype and personality traits in a large number of healthy Japanese subjects. However, these results should be regarded as preliminary and should be interpreted with caution because it is possible that the association between ABO blood group genotype and the Persistence trait is relatively weak. Further studies of this relationship between ABO blood type and personality traits could promote the understanding of human behavior and advance efforts to assess future risks of illness.

## Supporting Information

S1 Table (XLSX)
